# On the Cause and Better Treatment of Rickets

**Published:** 1907-06-08

**Authors:** William P. S. Branson

**Affiliations:** Assistant Physician, East London Hospital for Children


### Diseases of Children.

### ON THE CAUSE AND BETTER TREATMENT OF RICKETS.

By WILLIAM P. S. BRANSON, M.D., M.R.C.P., Assistant Physician, East London Hospital for Children.

In common with a number of other diseases, rickets has a well-known pathology, and a cure whose main lines are generally accepted, but a cause which continues to elude exact identification. It may be argued that under these circumstances the cause commands no more than an academic interest; but hardly with justice, for lack of particular knowledge on the subject makes treatment random, even if it be effectual, and slower in its operation, one may suspect, than would be the case did we know for certain the precise fault which calls for remedy. I propose to submit a few considerations, based upon clinical experience, which appear to me both to narrow the debatable issues of the matter and to indicate more direct avenues for successful treatment than those habitually followed at the present time.

### Causative Factors.

The causes of rickets, as given in the most recent text-books, are these. Generally unhygienic conditions of life, in particular lack of light and air, associated with a diet rich in carbo-hydrates, but poor in fats and proteids. Defined more strictly this proposition admits of the following interpretations, or combinations of these influences. Rickets (assuming the predisposing causes) depends upon (*a*) deficiency of (1) fats, (2) proteids, or in the alternative, (*b*) excess of carbo-hydrates, particularly starches, fats, and proteids being sufficiently ample. As regards the effect upon the growing organism of an absolute deficiency of fats, there is experimental evidence that young pigs fed upon milk from which practically all fat has been removed do not become rickety, although they become very thin.[1] Similarly, that mere lack of proteids is insufficient to produce rickets seems to be demonstrated by the fact that young children may die from slow starvation, either as a result of neglect, or of gastro-intestinal disorders, without evincing signs of rickets. We must give a more detailed consideration to the third alternative, namely, that rickets is due to an excess of carbo-hydrates, the other food elements being present in quantities sufficient to preserve health apart from such excess.

The commonest victims of rickets are children of the poor in the second and third years of life. From inquiries made at the East London Hospital for Children it appears that the standard dietary for children at this age and in this rank of life has some such basis as follows, subject of course to incidental variation from time to time. *Breakfast*: Bread and butter, or bread and dripping, with weak tea. *Dinner*: Bread and butter, or bread and dripping, pudding, either suet pudding or, less often, "milky pudding" of rice or sago. (The British matron affects suet pudding, the Jewish matron sago. I have not observed any notable predominance of rickets in either community at these later ages, although during the first year of life Jewish children seldom suffer from rickets. This is due to the almost universal custom of breast-feeding among the Jews.) *Tea*: Bread and butter or bread and dripping. Milk holds a very subordinate position, and most mothers of this class consider meat to be an improper article of consumption for young children, though fat bacon is often given.

### Errors of Diet.

The above dietary was lately recounted to me by the mother of a fat though dwarfed and rickety boy of four years, and is typical of many. Reviewing in the light of it the possibilities of fault already detailed it would appear that, monotony notwithstanding, such a bill of fare contains in ample quantity all the necessary elements of diet. Nor can it be said that co-existing errors of assimilation stultify the supply, for, common as are gastro-intestinal disturbances among rickety children, the disease is quite often met with in subjects who show no signs of digestive disorder, and who, judged by ordinary standards, assimilate well. It seems that of our possibilities the one which is certainly fulfilled is (*b*), that, namely, which asserts that rickets is due, not to an absolute deficiency in any element of diet, but to an excess of carbo-hydrate. This I believe to be the true explanation of rickets: that it is due to the disproportionate stimulation, by an excess of carbo-hydrates, of certain items of development, and represents an uneven advance of the line of growth. Vague as these words are, I cannot better them at present.

This view is strongly supported by such a case as the following: A child of well-to-do and careful parents came under my notice at the age of 18 months for the reason that no teeth had yet been erupted. He was fat but rather anæmic, and presented well-marked evidences of rickets in beaded ribs and enlarged epiphyses. I was credibly assured by his mother that he had consumed approximately two pints of cow's milk, obtained from a good dairy, daily for months past, but had in addition eaten largely—for he had a good appetite—of bread and butter, and of rice and other "milky puddings." It is incredible to me that this child had received an insufficiency of any of the elements of a healthy dietary, and there was nothing about him to suggest a faulty assimilation of food in general. But he certainly had eaten much farinaceous material. Within two months of the institution of a practically starchless diet the teeth began to appear in rapid succession, and the other rickety manifestations entirely vanished.

### A Few Points in Treatment.

A second example of almost equal force is supplied by a child lately attending the casualty department of St. Bartholomew's Hospital, who at the age of nine years presented all the signs of continued and active rickets. She was dwarfed, knock-kneed, and very weak, while the epiphyseal enlargements of rickets were extremely prominent. She was placed upon a starchless diet for six months, and was kept from walking by the application of splints. At the expiry of this interval I was disappointed to find that I could detect no improvement, and referred her to the Orthopædic department to see whether surgery could benefit the deformity of the legs. Here a skiagram of the bones was taken, and contrary to my expectation, the epiphyses showed no signs of active disease. The epiphyseal line was natural in spite of the continued enlargement of the ends of the bones. The splints were removed, and under treatment by massage a rapid improvement of the general condition ensued. There can be no doubt I think that the prolonged reduction of carbo-hydrates produced the cure.[2]

The correct treatment of rickets lies therefore in the proper adjustment of the carbohydrate supply. This can be achieved in two ways, either by levelling up the fats and proteids to correspond with the high carbo-hydrate intake common in the case of rickety children, or by levelling down the carbo-hydrates. I have little doubt that the latter is the wiser plan, though the other is that more commonly employed. Children thrive well on a diet poor in carbo-hydrates provided proteids and fats are well represented, while the levelling-up method involves the risk of over-taxing the digestion. For a rickety child of two years the following is a suitable daily menu, if digestion is good. Milk, two pints, with the occasional addition of some malted food such as Mellin's. A half-ounce or an ounce of scraped beef or fish, or an egg. A baked apple or some orange-juice, with a teaspoonful of cod-liver oil and malt given three times a day.

[1] "Journal of Experimental Medicine," 1898, iii. 293. [2] This case, with other examples of continued rickets is reported by Mr. R. C. Elmslie, St. Bartholomew's Hospital Reports, 1906, p. 155.

